# Wine Traceability: A Data Model and Prototype in Albanian Context

**DOI:** 10.3390/foods5010011

**Published:** 2016-02-17

**Authors:** Kreshnik Vukatana, Kozeta Sevrani, Elira Hoxha

**Affiliations:** Department of Statistics and Applied Informatics, Faculty of Economy, University of Tirana, Rruga e Elbasanit, Tirane, 1001, Albania; kozeta.sevrani@unitir.edu.al (K.S.); elirahoxha@yahoo.com (E.H.)

**Keywords:** wine data model, traceability information system, product chain, food safety, real-time notification

## Abstract

Vine traceability is a critical issue that has gained interest internationally. Quality control programs and schemes are mandatory in many countries including EU members and the USA. Albania has transformed most of the EU regulations on food into laws. Regarding the vine sector, the obligation of wine producers to keep traceability data is part of the legislation. The analysis on the interviews conducted with Albanian winemakers show that these data are actually recorded only in hard copy. Another fact that emerges from the interviews is that only two producers have implemented the ISO (International Organization for Standardization) standards on food. The purpose of this paper is to develop an agile and automated traceability system based on these standards. We propose a data model and system prototype that are described in the second and third section of this work. The data model is an adaption along the lines of the GS1 (Global Standards One) specifications for a wine supply chain. The proposed prototype has a key component that is mobile access to the information about wine through barcode technology. By using this mechanism the consumer obtains transparency on his expectations concerning the quality criteria. Another important component of the proposed system in this paper is a real-time notification module that works as an alert system when a risk is identified. This can help producers and authorities to have a rapid identification of a contaminated product. It is important in cases when recalling the product from the market or preventing it from reaching the consumer.

## 1. Introduction

Nowadays, consumers and winemakers show a growing interest in finding different ways to verify the genuineness and authenticity of their products. In this direction, a risk management tool such as a traceability system can be used by the consumers to easily trace the origin and the overall process of making a product. Implementing a food traceability system implies safer products. Such a system enables the tracing back through the food chain process. Under these conditions, food business operators or authorities can withdraw or recall products that have been identified as unsafe. For example, when an event such as a foodborne accident or food safety non-conformance happens, the system assists the operators in searching for its cause. In order to withdraw and recall a food with problems in an accurate and prompt way, the traceability system can narrow down the search for the said food as well as identify its destination. Then, if the food history information records are maintained, the system makes it easier to collect data about unexpected and long-term impacts on human health attributable to food history. Furthermore, the traceability system can help in developing risk management measures and clarifying responsibilities of food business operators.

### 1.1. Policy Requirements

European Council (EC) regulation No. 178 defines traceability as “the ability to trace and follow a food, feed, food-producing animal or substance intended to be, or expected to be incorporated into a food or feed, through all stages of production, processing and distribution” [1] (p. 8). In relation to the traceability of wine, the EU has adopted rules and provisions in three key areas, which are: (i) quality and additives used to make different types of wine; (ii) labeling; and (iii) record-keeping [2,3], including the documentation required to certify the authenticity of wines being transported in bulk containers [4].

Concerning the ISO (International Organization for Standardization) standards, ISO 22000 [5] specifies requirements for a food safety management system, where an organization in the food chain needs to demonstrate its ability to control food safety hazards, in order to ensure that food is safe at the time of human consumption. In it are incorporated the principles of the CAC’s (Codex Alimentarius Commission) “Hazard Analysis and Critical Control Point, HACCP” system for food hygiene [6]. ISO 22005 is the latest in a series of food safety standards launched in 2005 [7]. It uses the same definition of traceability as the Codex Alimentarius Commission [8] and provides a complement for organizations implementing the ISO 22000:2005 standard. It gives the basic requirements for a food safety management system to ensure safe food supply chains.

In Albania, the requirements for traceability in viticulture are included in the regulations that follow law No. 9863 [9]. These requirements lean on the general food law, EC regulation No. 178, including a list of requests for food traceability. In particular, article 25 of this law lists the requests for a system of self-control, which is based on the HACCP (Hazard Analysis and Critical Control Point) definition. Winemakers are obligated to register the needed information and prepare annual reports that are stored in regional departments of the Ministry of Agriculture. The information can be preserved in hard copy or electronic copy, but there is no obligation to choose one method over the other.

### 1.2. Logistics in Traceability

A number of traceability systems, technologies and standards have been developed to carry out supply chain traceability, with different business objectives [10,11,12,13]. These systems can be as simple as recording the batch codes of ingredients at each stage of production, or as sophisticated as computerized bar-coding to track and control the movement of ingredients and finished goods. In the automation of supply chain traceability, some standards and technologies gained a leading role. Radio-frequency identification (RFID) [14] and Electronic Product Code (EPC) global [15] are considered to be the most appealing sensing technologies and paradigms for supply chain traceability. EPC global can be implemented in both RFID and two-dimensional (2D) barcode technologies. The RFID system can be integrated with a Wireless Sensor Network (WSN), where an Internet application can therefore be used to monitor and gather information from tags. An example is shown in the work of Anastasi *et al.* [16], where a system for monitoring the productive cycle of a Sicilian winery is implemented, in which sensor nodes are deployed both in the field and in the cellars where the wine aging occurs. Meanwhile, 2D barcodes such as QR (Quick Response) codes can store a sufficient amount of data. They have a good readability, even on small-sized labels, and also in cases of physical damage of a part of the code. Compared to RFID tags, 2D barcodes need a line of sight. Cunha *et al.* show a system for vineyard identification and vine origin based on a QR code printed on the container where the vine is transported. By reading the QR code with a mobile phone and with Internet access, a user can get information about the origin, weather and other conditions in the field during the growth of the grapes [17].

Regarding the ISO standards, Global Standards One (GS1) is one of the best-known developing groups. It is an international non-profit association, dating back to an *ad hoc* group that came together to develop uniform grocery product codes. GS1 has published a document on wine supply chain traceability based on EC regulation No. 178 [18]. This document provides the recommendations and guidance needed to understand and implement the GS1 system of numbering and bar-coding from the grape grower to the retailer. Table 1 shows the key areas with the information that has to be recorded.

The data described in Table 1 are a starting point for the modified data model introduced in Section 3.1. This transformation of the GS1 data model is necessitated by the Albanian context, where only three areas are easily identifiable: grape grower, retailer and wine producer. The last-mentioned incorporates in its process the other key areas, such as bulk distributor, transit cellar, filler/packer and finished goods distributor.

### 1.3. Traceability-Driven Issues in Food Supply Chain Management

In their review, Dabbene *et al.* list four issues concerning traceability systems in food supply chain management [19]. The first is food crisis management. When a recall of a product is needed, the system must provide as much detailed information in a short amount of time possible for both processes: the backward identification of potentially deficient lots and the forward identification of potentially affected products that have to be withdrawn [20]. The work of Wynn *et al.* is a good starting point on how the information will be prepared, and in identifying common data requirements for traceability and data exchange, needed for the automation of the notification process in case of a recall [21]. The second issue to be considered is the traceability of bulk products. This is important in wine-making companies which use ingredients that are liquids. What happens is that, in many cases, they must be stored in huge silos which are rarely completely emptied and cleaned. This means that residues of preceding lots are merged partially with the lot in process, leading to the introduction of the concept of fuzzy traceability. By introducing a threshold, new virtual batches are then generated. Comba *et al.* developed the concept where the definition of a lot given in the ISO Standard 22005/2007 is rigorously formalized [22]. Quality and identity-preservation is another issue concerning the traceability systems. Product information regarding sensory, health, nutrition, *etc.*, which is part of quality systems, can be linked with traceability systems in order to enhance quality. The concept of identity preservation (IP) refers to the ability to maintain particular traits and/or attributes [23]. Attributes such as country of origin, organic, *etc.*, which are not necessarily dependent on quality characteristics, can be included in IP to increase the value of the product as perceived by the consumer. The final issue is fraud prevention and anti-counterfeit techniques. These technologies for food authentication can be paired in the traceability systems [24,25,26,27] and are implemented in both RFID tags and barcodes.

### 1.4. Objectives

The objective of this paper is to develop an agile and automated traceability system using electronic traceability data, given the context for communities of small-scale enterprises. The proposed system tries to solve all the above issues related to traceability in supply chain management. The interviews conducted with companies that produce wine in Albania show the lack of such a system. This observation may negatively affect the recall process. Developing the prototype suggested in this paper helps the authorities in accelerating the identification process by finding the exact location and the precise date/time of the product that must be recalled. This means that each company, which is responsible for documenting where the goods come from, where they go, and with which processes they are treated, has the opportunity to do it with less effort through the system. The possibility for the consumer to easily access targeted content and information about the product in real time is another enhancement offered by the proposed system. The goal is to realize an integrated system of information which enables traceability management in wine products based on the data model we propose, with the possibility of extending it to any company in the food sector.

## 2. Materials and Methods

Using a web-based system for data processing, storage and exchange offers very easy access to the information. Integrating the system with mobile and web applications prepares system usability for all kinds of users. Moreover, it allows a real-time monitoring of the traceability process. Setting the system in Service-Oriented Architecture lets it link different data sources and functionality [28]. The encrypted data carriers such as encrypted barcodes limit the possibility of counterfeiting the data related to the product and allow it to be easily differentiate it from a fake one [27].

### 2.1. Web and Mobile Application

The prototype presented in this paper is based on a three-tier architecture. It is a client-server software architecture pattern where the user interface (presentation), functional process logic (“business rules”), data storage and data access are developed and maintained as independent modules, most often on separate platforms. For the presentation layer, two types of applications are used (web and android), both written in the Java language. Some of the factors affecting the selection of web and mobile technologies in our system are: the displaying of data in a fast and user-friendly way, the mobility of operators, the ease of reading and tagging, and the wide spread usage of those technologies.

### 2.2. Encrypted Barcodes

Barcodes are basically data carriers associated with the process of labeling a product. They are used in the overall process from the producer to the consumer for different tasks as storage, producer ID, sales receipts, *etc.* We have chosen this technology for our system instead of RFID because barcodes are inexpensive to design and print. Their fonts can be downloaded from the Internet, often for free, and can be customized economically in a variety of finishes and materials. In our case, wine bottles already have a label, so printing the barcode on this label will not increase the cost of the product and also the traditional labeling would be preserved. Another reason for choosing barcode technology over RFID is that many smartphones now include applications that scan and interpret barcodes. These kind of applications can also be downloaded for free from various sources. The barcode approach is user-friendly; lately, most of the product labels already have barcodes, so the consumer acceptance for this method of getting traceability data is almost guaranteed. Our research is focused on the QR code as part of the second generation of barcodes known as 2D barcodes. According to US Mobile Marketing [29], the growing trend of spending on mobile recognition ought to almost double in 2014. This trend continues to grow strongly, and the estimate is based on reports of increased scanning levels. ScanLife, which is one of the global leader companies providing cloud-based mobile solutions and QR code technologies, stated that it processed 18 million scans via its ScanBuy application in Q1 of 2013. That is up from 13 million scans in Q1 of 2012, which in itself was up 157% over Q1 of 2011, as shown in the Marketing Charts report [30]. Kato and Tan show that QR codes are mostly used through 2D barcodes integrated in camera phone applications [31]. In their analysis they benchmarked both the database-based codes (QR and Data Matrix) and index-based codes (Visual Code, ShotCode, and ColorCode). Also, Jackson stresses this fact in his research on smartphone users [32]. The idea of preventing counterfeiters from having the same traceability as the original product is done by introducing the use of encryption, where a cryptographic algorithm such as RSA (Rivest-Shamir-Adleman cryptosystem) is used for having an encrypted QR barcode.

### 2.3. Web Services and Database Modules

**Figure 1:**
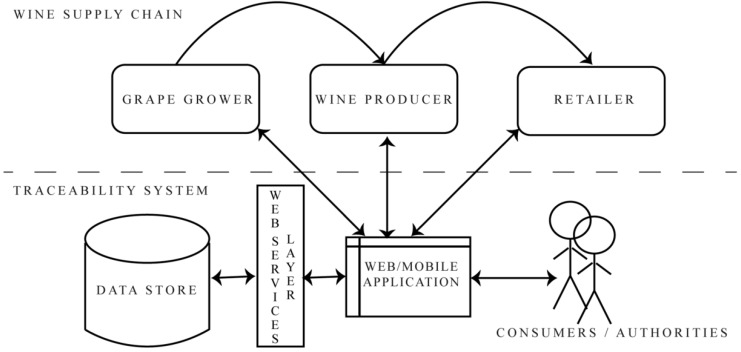
The proposed prototype. The figure shows the wine supply chain in the Albanian context, integrated with an automated traceability system.

A Service-Oriented Architecture (SOA) is built on top of the prototype architecture. Implementing such architecture leads us in the direction of having fully decoupled, service-based computing [33]. This new approach allows faster delivery of applications, less coupling, and better scalability. A web services layer is integrated with the relational database, with the traceability data acting as an interface, as shown in Figure 1. The communication between applications and the database is done through messages in XML. The partitioning of the database in modules that represent the data model inducts a better interoperability between the web services and the modules themselves.

## 3. System Proposal

To better understand the situation of wine traceability information and how these data are collected, interviews were conducted with some Albanian winemakers. Questions on ISO standards and how they are implemented were asked as well. The selection of wine-making companies to be interviewed, six in total, was made based on their production history and the market demand for their products. The interview was divided into two parts. The first part contains questions about the phases of wine production that are recorded for traceability, including vineyards, fermentation, aging, maintenance/finishing, and conditions of the final product. Meanwhile, in the second half of the interview, questions about GS1 standards are formulated on issues such as identification and labeling of products, and scanning capabilities combined with electronic information flow and data recording. The interviewers were asked to evaluate their company’s level of implementing the GS1 standards with a score from 0 to 4 as follows: (0) no action taken; (1) plans have been established but the work has not started; (2) implementation has started with a limited scope (e.g., some product categories); (3) rollout of the full implementation has started; (4) plans fully implemented. 

The information collected gives rise to an adaption along the lines of the GS1 data model. Furthermore, the scheme of the wine-making process (Figure 1) is adapted from the traceability system architecture proposed by Vukatana and Hoxha to fulfill the requests based on the new data model [34]. From the specifics of the environment and the vines to the harvest, finished wine and delivery, each step matters. Implementing a traceability system will help to ensure that each step taken is a step toward a higher quality by monitoring viticulture data; tracking farming and producing activities; and enriching the data store with important information in the interest of all the stakeholders (business operators, authorities and the final client).

### 3.1. Data Model

**Figure 2:**
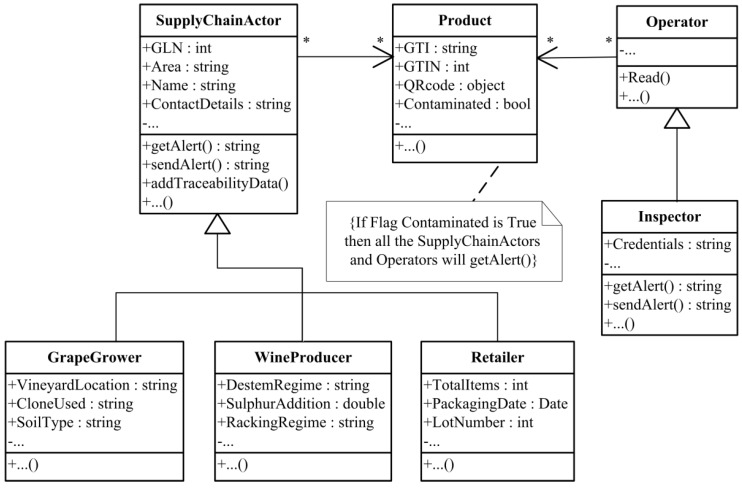
UML class diagram. The figure shows the wine traceability data model.

In order to assess the fundamental requirements of wine traceability, a data model is proposed (Figure 2), based on the wine supply chain defined by the working group of GS1. This standard model is changed slightly to adapt to the Albanian reality. The five actors determined by GS1 standards (Wine Producer, Bulk Distributor, Transit Cellar, Filler/Packer, Finished Goods Distributor) are included in only one key area which is the Wine Producer to better represent the real scenario in Albania. This happens because the wine producers in Albania cover all the processes of the other four actors mentioned above and all the needed information for the traceability chain of the four areas is provided by this category. The two other main actors present in the architecture of our system are the Grape Grower and the Retailer. Each actor is responsible for specific activities which have to be traced in order to enable supply chain traceability. These activities and the corresponding data which have to be collected to make traceability effective are described in Table 2.

The data model must be general enough to represent all these traceability elements and their activities managed within a wine supply chain, such as material application management, fertilizer types, irrigation management, parcel identification and grape monitoring in the vineyards, process monitoring and steering in wine cellars, *etc.* The model will also have to record the relation between these activities and other additional data on the product quality such as humidity and cellar temperature history. We used the Unified Modeling Language (UML) to denote this model. Figure 2 shows a representative part of the traceability data model class diagram, where there are the main classes and their associations.

Every product should be uniquely identified. In order to overcome problems of confusion, duplication and misinterpretation, a solution based on the approach of the GS1 numbering system which provides global uniqueness is adopted. Each actor in the supply chain will be uniquely identified by the numeric code GLN (Global Location Number) assigned to any legal entity. 

Thus, a Wine Producer with its own GLN can be supplied from two different Grape Growers that are identified by GLN_x_ and GLN_y_, and can distribute its production to several Retailers each of which have a different GLN. Further, the system will assign a unique identification number to any trade item made by the wine producer called the GTIN (Global Trade Item Number).

Another number used by the GS1 standard is the SSCC (Serial Shipping Container Code) which uniquely identifies the logistic units for transport and/or storage. Trade units and logistic units can be associated with attribute information such as batch number, production date, *etc.*, and for this reason another identifier is added for every attribute and is called AI (Application Identifier). The four mentioned identifiers are then combined in a single code that will accompany every bottle of wine. We call this code the global traceability identifier (GTI) because it will be used by every actor of the supply chain to add traceability data to the product, and also by the authorities and final clients to get this information.

### 3.2. Prototype Description and Functionalities

Papetti *et al.* developed the concept of infotracing for a typical Italian cheese, where product traceability data are used to classify the products [35]. This classification is based on the product quality analysis (chemical, sensorial and spectrophotometric) at the end of the product lifecycle and uses all the parameters related to the maturation phase of the cheese. Data collection in this kind of system is done automatically by using active RFIDs. The idea of infotracing can also be implemented in our system by adding information in a unique, final traceability certificate. In our case this information will be added manually from each supply chain actor. For example, the grape grower will add the harvest dates for the vines, the historic temperature, *etc.*; the wine producer will register information on fermentation, type of barrels, post-fermentation, cellar temperature, *etc.*; and the retailer will add information on location, batch number, *etc.* The lower part of Figure 1 shows the interaction between the actors and the system, and a detailed list of the attributes that each actor is responsible for registering in the system is shown in Table 2. The way each supply chain actor accesses the system to add the data for a specific product is as follows: The GTI representing the product is generated by the web application and is used to access the associated traceability data. This identifier initially is encrypted by the system, then it is coded, and the result (a QR code image) is given to the producer to be included on the bottle label. The same generated unique QR code is given to the three actors in order to access the system to enrich the product traceability certificate.

The encryption protects consumers from third parties that want to launch a counterfeited product with an existing identifier in the market. Food operators can access the system to add or inspect traceability data through a web/mobile application which has a decryption key which recovers the GTI of the product. The system based on encrypted barcodes allows two principal security roles: operators (business and authorities) and consumers. The business operators in the food chain such as crop farmers, wine-makers or retailers can modify traceability data. Authorities (e.g., food control inspectors), on the other hand, can access the system in order to verify through traceability if a product is either counterfeited or meets the requirements. The second group, the consumers, can access in a read-only mode all the information about a food product through the software on their smartphones.

The system is based on real-time processing in order to gather and process data from different stakeholders fast enough so that the results are visualized at the right time. The conceptualization of the system in this way helps to monitor and control a large number of distributed events of interest by collecting and processing raw data that will be stored in the database. The application will reflect the current state of the environment and will be updated frequently to make a realistic model. A real-time notification is another important component of the proposed system. It works as a rapid alert system when a risk is identified. Thus, each one of the supply chain actors can signal through the application when they find a contaminated product or a product that does not comply with the standards. In this moment the responsible actors are notified and the product identified as unsafe can be withdrawn from the market or can be prevented from reaching the consumers.

## 4. Discussion

The discussion deals with two crucial points. First, are there records about traceability for wine products in Albania? Second, are wine-making companies interested in integrating a traceability system into their product chain? To address the first question, a study on the Albanian legislation was taken. Albania obtained EU candidate status in June 2014 and EU membership is one of the major objectives of the government. This goal is associated with various reforms, which are implemented in different areas, and food is one of them. From this aspect, the traceability process is part of the Albanian legislation, and recording traceability data for the wine-makers is obligatory. The interview mentioned in the data model section was made to check how the mechanism of recording traceability data is implemented by the companies. The questions asked to all the wine-makers were the same. The answers of the interviewers bring out the fact that the data are recorded, but in the most of those companies the collected information is stored in hard copy at regional departments of the Ministry of Agriculture. When an incident occurs, the current method of data storage is not very efficient. The ability to trace the food back to its origins can be crucial for the government and industry during a food-related recall or outbreak. Setting up a traceability system which handles real-time processing of data such as location and time implies a reduction of the response time. In this manner the process of identification for the product and its withdrawal from the market is accelerated.

The interview included, in addition, questions about the standards applied by the companies in the food chain. Two of the companies interviewed were certified by ISO 22000:2005. The ISO certificate means that the requirements for a food safety management system are respected. An organization in the food chain needs to demonstrate its ability to control food safety hazards in order to ensure that food is safe at the time of human consumption. The implementation of the proposed prototype facilitates the efforts for the companies that are not certified to become certified by these ISO standards. This is due to the fact that the system is based on the GSI recommendations, which implement the best practices to achieve compliance with EC regulation No. 178.

To address the second question, each company during the interview was shown the functionalities of the system and how it is integrated within the food chain process. These functionalities are able to quickly identify the product, which lots are involved, where they are shipped and where are they now, *etc.* The wine-makers show interest in integrating the proposed prototype in the food chain due to the low cost, which is transposed in adding a barcode to the labeling process. Furthermore, with regards to the consumers, the traceability system gives them transparency—the ability to trust that they know what they are drinking. Companies that build this kind of relationship with their customer, will most likely have a rapid gain in the market share. 

## 5. Conclusions and Future Work

This paper shows the actual situation of traceability data in the vine sector in Albania, through analyzing the information handled by the wine producers. It describes the application of standards in the wine supply chain and the steps that have to be taken toward the overall process of tracing. The lack of a traceability system in this sector led us to propose a data model and a system prototype that uses information and communication technologies with focus on traceability. The system is based on mobile and web applications, and operates by integrating web services and encrypted QR codes. It complies with GS1 standards, thereby facilitating the efforts of companies to be certified by ISO standards. Owing to its ease of use, it can be efficiently utilized by all the stakeholders, from wine-makers to authorities and consumers. This kind of approach leads to safer and better products and more transparent processes.

However, a lot of work has to be completed in order to build the whole system and the overall processes that comprise the proposed architecture. Real data from the Albanian wine supply chain will be used for testing the implemented prototype. These data will cover the complete processes through which the production of a bottle of wine passes until it is delivered to the retailer. The execution of the test cases and the evaluation of the respective results by means of quality and quantity measures will check the correctness, completeness and performance of the developed prototype.

## Figures and Tables

**Table 1:** The key areas in the wine supply chain determined by the GS1 (Global Standards One) working group [16]. Each area is associated with relevant GS1 standards to be deployed.

Key Areas	Specification of Attributes
Grape Grower	Name and address of the vineyard, plot map reference/cadastral reference/block identifier, size of plot/number of vines, vine variety and contact details.
Wine Producer	Identification of the wine producer, product identification, shipping container identification, the quantity of wine dispatched and the batch number of each product.
Bulk Distributor	Identification of the bulk distributor and identification of the bulk wine container associated with serial shipping container code, global trade item number, batch and quantity numbers.
Transit Cellar	Identification of the transit cellar, identification of a container, product identification, the quantity of wine dispatched, and the batch number of each product.
Filler/Packer	Shipping container identification, identification of filler/packer and lot number of the shipped items; link these to the identification of location of the recipient.
Finished Goods Distributor	Shipping container identification of the inbound pallet and identification of location of its supplier, shipping container identification of the outbound pallet, either unmodified or newly created and identification of the retail.
Retailer	Logistic units (pallets) are identified by items contained, count of the items contained, packaging date and batch/lot number.

**Table 2:** The key areas in the wine supply chain adopted in the context of Albanian wine-making companies. Each area is associated with the information to be traced.

Key Areas	Specification of Attributes
Grape Grower	Information on vineyard, such as location, clone used, space of vines for acre, age of vines, type of soil and drainage, irrigation, type of water used, harvest dates, lot identification, brix, Ta, pH, temperature historic recording, *etc.*
Wine Producer	Information on fermentation such as crush/destem regime, whole berry component, % of stems returned to fermenter, sulphur additions at fermenter, pre-fermentation maceration days, fermenter sizes, acid/sugar adjustments, yeast inoculum, has nutrients/other additions, temperature history/controls records, punch/tread/pump over/irrigate regime, primary duration, lactic bacteria inoculum, if done, when, post-fermentation maceration duration, press type and regime and pre-barrel settling time *etc.* Information on aging such as type of barrel, age, % of blend and expected time in oak. Information on maintenance and finishing such as cellar temperature history, racking regime, heat and cold stabilization planned, expected filtrations and expected final additions. Information on final product such as % of alcohol, Ta, pH, SO2 and RS, lot number and distribution records.
Retailer	Information on location, count of the items contained, packaging date and batch/lot number

## Abbreviations

The following abbreviations are used in this manuscript:

ISO: International Organization for Standardization
GS1: Global Standards One
CAC: Codex Alimentarius Commission
HACCP: Hazard Analysis and Critical Control Point
RFID: Radio-Frequency IDentification
EPC: Electronic Product Code
QR: code: Quick Response Code
RSA: Rivest-Shamir-Adleman cryptosystem
SOA: Service Oriented Architecture
XML: EXtensible Markup Language
UML: Unified Modeling Language
GLN: Global Location Number
GTIN: Global Trade Item Number
SSCC: Serial Shipping Container Code
AI: Application Identifier
GTI: Global Traceability Identifier
Ta: Titratable Acidity
RS: Residual Sugar
